# Evaluation of conditional survival outcomes in patients with redefined anaplastic thyroid carcinoma

**DOI:** 10.3389/fendo.2025.1525869

**Published:** 2025-06-10

**Authors:** Yinuo Zheng, Yizhen Zhuang, Peng Zhou

**Affiliations:** ^1^ Department of Thyroid and Breast Surgery, The Third Affiliated Hospital of Wenzhou Medical University, Wenzhou, China; ^2^ Department of Medical Record Office, The Third Affiliated Hospital of Wenzhou Medical University, Wenzhou, China; ^3^ Department of Heria and Abdominal Wall Surgery, The Third Affiliated Hospital of Wenzhou Medical University, Wenzhou, China

**Keywords:** SEER, anaplastic thyroid carcinoma, conditional survival, nomogram, dynamic survival prediction

## Abstract

**Objective:**

Conditional survival (CS) offers a more precise assessment of prognosis by continuously updating to reflect the current state. This study aimed to analyze the CS outcome of redefined anaplastic thyroid carcinoma (rATC).

**Methods:**

A cohort comprising 1424 patients diagnosed with rATC between 2000 and 2018 was extracted from the Surveillance, Epidemiology, and End Results database and subsequently divided into training and validation sets at a ratio of 3:1. We analyzed the CS prognosis of these patients. A Lasso Cox regression model was employed to determine independent risk factors for long-term survival and to develop a predictive nomogram integrating CS analysis, aiming to provide CS estimates and stratify the risk among rATC patients.

**Results:**

The likelihood of achieving 5-year CS escalated from 8% after diagnosis to 44%, 68%, 82%, and 95% after 1, 2, 3, and 4 years of survival, respectively. As patients endured longer, their probability of further years of survival augmented. The Lasso Cox regression analysis identified age, tumor stage, surgery, radiotherapy and chemotherapy as the most influential prognostic factors associated with outcomes. Ultimately, we successfully established a novel CS-nomogram, aiming to provide dynamic survival updates for these patients. Our predictive model can quantify patients’ risk and stratify it accordingly. Furthermore, after evaluation, the performance of our predictive model was found to be satisfactory.

**Conclusion:**

Utilizing extensive SEER datasets, we devised and validated a CS-nomogram, enabling the prediction of the conditional probability of survival for rATC patients. This facilitates the incorporation of survivorship duration into prognostic assessments.

## Introduction

Anaplastic thyroid carcinoma (ATC) stands out as among the most aggressive and lethal solid malignancies, constituting only 1%-2% of thyroid cancers (1–3). Despite its rarity, it portends a bleak prognosis, contributing significantly to 50% of all thyroid cancer-related mortality (1, 4). In contrast to the typically favorable prognosis in differentiated thyroid cancer, patients with ATC commonly present with rapidly proliferating and infiltrative neck masses, accompanied by regional cervical lymph node involvement (5). Additionally, around half of these patients also demonstrate distant metastatic spread (5–7). Primary squamous cell carcinoma of the thyroid (PSCCTSH) is another exceptionally rare and deeply invasive malignancy, now classified as a morphological variant of ATC in the fifth edition of the WHO Classification of Endocrine and Neuroendocrine Tumors (8, 9). Currently, there is a scarcity of research comprehensively analyzing the survival outcomes and prognostic features of redefined anaplastic thyroid carcinoma (rATC), which encompasses both ATC and PSCCTH, within a sizable patient cohort.

Additionally, most survival rates reported in the literature are static, often calculated from the day of diagnosis or surgery. However, the life expectancy of cancer patients often changes with increasing survival time (10, 11). Generally, as survival time lengthens, life expectancy of cancer patients gradually extends, approaching that of the general population. Conditional survival (CS) analysis, as an innovative approach in bioinformatics, incorporates the patient’s existing survival time to dynamically estimate the probability of additional survival time for cancer patients (12–14). In contrast to traditional survival analysis, which only provides fixed survival probabilities at different time points, CS analysis offers real-time, dynamically updated prognostic information. Indeed, with the development of more valuable treatment regimens and follow-up protocols for cancer patients, this approach will offer deeper insights into validating treatment effectiveness and optimizing follow-up strategies.

In addition to the time elapsed since diagnosis, factors such as clinicopathological features also play a significant role in determining the likelihood of survival. Nomogram models, widely employed in oncology, utilize patients’ clinicopathological characteristics to quantify prognostic information (15–17). However, they lack consideration for the duration of a patient’s survival, thus compromising their precision. For instance, traditional nomogram models fail to provide real-time updates for patients who have survived for a certain number of years.

While traditional prediction models have been established for rATC in existing literature (18–20), the integration of CS into a nomogram format remains to be developed. Therefore, in this study, we utilized the Surveillance, Epidemiology and End Results (SEER) database, which provides a large sample cohort for rare tumors, to analyze the CS prognosis of rATC patients. And we also established a corresponding nomogram model integrated with CS analysis. Finally, we attempted to implement risk stratification using this model, thereby facilitating prognostic management and follow-up monitoring for rATCs.

## Methods

### Data collection

SEER-State software (version 8.4.2; http://seer.cancer.gov) was employed to retrieve rATC data from the SEER database. The study’s inclusion criteria comprised: (1) Histology coded based on ICD-O-3, specifically 8012/3, 8020/3, 8021/3, and 8030/3–8032/3 for ATC, and 8070/3–8076/3 for PSCCTH; (2) Tumor site coded as C73.9, indicating the thyroid; and (3) Diagnosis falling within the period of 2000-2018. Exclusion criteria encompassed: (1) Concurrent presentation with other malignancies; (2) Diagnoses solely based on autopsy findings or death certificates; and (3) Incomplete variable data, with tumor size excluded. This study was approved by the Ethics Committee of the Third Affiliated Hospital of Wenzhou Medical University.

### Definitions of variables and endpoints

Variable definitions and SEER database-derived information encompassed patient age at diagnosis, sex, ethnicity, histology, tumor stage, tumor size, surgical procedures, radiotherapy, chemotherapy, marital status, and household income. We defined variables based on the SEER Program Coding Manual and official documentation. Tumor stage was categorized as locoregional or distant. Locoregional stage refers to tumors confined to the thyroid or those that have spread to regional lymph nodes or adjacent tissues, while distant stage indicates metastasis to remote organs (e.g., lungs or bones). Marital status, classified as a demographic variable, was recorded as the marital status at diagnosis, typically categorized as single or married. For treatment-related variables, surgery was categorized as no surgery, subtotal/near-total thyroidectomy, and total thyroidectomy. In the SEER database, chemotherapy and radiotherapy are recorded as binary variables (Yes or No) due to limitations in data collection. Detailed treatment information—such as type, dosage, or duration—is not consistently available, limiting in-depth analysis but still allowing for a general understanding of treatment patterns in large population-based cohorts. OS referred to the duration in months from diagnosis until death from any cause for rATC patients at the conclusion of follow-up. The endpoint event was defined as death from any cause; instances where this did not happen were censored.

### Statistical analysis

The categorical variables were displayed as frequency (percentage). The OS was evaluated through Kaplan-Meier analysis, and the survival curves were compared using the log-rank test.

CS was defined as the likelihood of surviving an additional ‘y’ years, given that a patient had already survived for ‘x’ years. It was calculated using the formula CS(y|x) = S(y+x)/(x), where S(x) represents the OS at ‘x’ years estimated using the Kaplan-Meier method. For instance, the CS at 1 year following 4 years of survival (CS(1|4)) was determined by dividing the 5-year Kaplan-Meier OS estimate (S(5)) by the corresponding 4-year estimate ( S(4)).

All eligible rATC cases underwent random allocation into either the training or validation cohort, with a split ratio of 7:3. The training cohort was utilized for developing the CS-based nomogram model and establishing the risk classification system. Utilizing the least absolute shrinkage and selection operator (LASSO) analysis can effectively decrease model complexity, mitigate overfitting, and improve interpretability, especially in situations with high-dimensional datasets. Hence, we employed a penalized LASSO Cox proportional hazards model to screen prognostic variables, aiming to address overfitting concerns. Subsequently, the prognostic variables identified through screening were utilized to construct a nomogram model. Moreover, we incorporated CS into the nomogram, aiming to consider both the impact of clinical characteristics on prognosis and supplement the predictive role of CS probabilities. This approach will provide more precise and dynamic prognostic information for these patients with a pessimistic outlook, yielding deeper insights and offering crucial guidance for disease management and follow-up monitoring.

The nomogram’s predictive performance was assessed through the Concordance index (C-index) and the receiver operating characteristic (ROC) curve with the area under the curve (AUC) value. Calibration curves were utilized to gauge the nomogram’s accuracy. Furthermore, Decision Curve Analysis (DCA) was employed to evaluate the clinical net benefit capacity of the nomogram model. To further validate the applicability of our model, we included two ATC cases from our center (The Third Affiliated Hospital of Wenzhou Medical University)—given the rarity of ATC—and predicted their survival probabilities based on their clinicopathological characteristics.

Ultimately, the CS-nomogram was employed to calculate total points for each patient and determine optimal cutoff points for stratifying them into two risk-based groups. Subsequently, a Kaplan-Meier (KM) curve was generated for survival analysis.

All statistical analyses were conducted using R software. A significance level of p<0.05 (two-sided) was considered statistically significant.

## Results

### Demographic and clinicopathological characteristics

A total of 1424 patients diagnosed with rATC, comprising 1309 with ATC and 115 with PSCCTH, were identified in the SEER database between 2000 and 2018 (**Table 1**). These patients were then stratified into a training group (n=996) and a validation group (n=428). The majority of individuals in the entire cohort were over 60 years of age (74.7%), identified as white (78.7%), and female (60.5%) at the time of diagnosis. And the majority of patients (79.1%) were diagnosed with distant metastasis at the time of diagnosis. Furthermore, it was observed that 46.2% of the patients underwent surgical intervention, 57.4% received radiotherapy, and 44.4% underwent chemotherapy.

**Table 1:** Demographic and clinicopathological characteristics of rATC.

Characteristics	Overall	Training	Validation	P
	(N=1424)	(N=996)	(N=428)	
Age				0.493
≤60	360 (25.3%)	259 (26.0%)	101 (23.6%)	
61-70	373 (26.2%)	267 (26.8%)	106 (24.8%)	
71-80	410 (28.8%)	278 (27.9%)	132 (30.8%)	
>80	281 (19.7%)	192 (19.3%)	89 (20.8%)	
Sex				0.522
Male	562 (39.5%)	399 (40.1%)	163 (38.1%)	
Female	862 (60.5%)	597 (59.9%)	265 (61.9%)	
Ethnicity				0.628
White	1121 (78.7%)	787 (79.0%)	334 (78.0%)	
Black	118 (8.3%)	78 (7.8%)	40 (9.3%)	
Others	185 (13.0%)	131 (13.2%)	54 (12.6%)	
Tumor Histology				0.843
ATC	1309 (91.9%)	917 (92.1%)	392 (91.6%)	
PSCCTh	115 (8.1%)	79 (7.9%)	36 (8.4%)	
Tumor stage				0.229
Locoregional	426 (29.9%)	308 (30.9%)	118 (27.6%)	
Distant	998 (70.1%)	688 (69.1%)	310 (72.4%)	
Tumor size				0.06
≤40mm	224 (15.7%)	170 (17.1%)	54 (12.6%)	
41-60mm	310 (21.8%)	224 (22.5%)	86 (20.1%)	
>60mm	577 (40.5%)	378 (38.0%)	199 (46.5%)	
Unknown	313 (22.0%)	224 (22.5%)	89 (20.8%)	
Surgery				0.222
No surgery	766 (53.8%)	521 (52.3%)	245 (57.2%)	
Subtotal/Near-total Thyroidectomy	296 (20.8%)	212 (21.3%)	84 (19.6%)	
Total Thyroidectomy	362 (25.4%)	263 (26.4%)	99 (23.1%)	
Radiotherapy				0.39
No	606 (42.6%)	416 (41.8%)	190 (44.4%)	
Yes	818 (57.4%)	580 (58.2%)	238 (55.6%)	
Chemotherapy				0.224
No	792 (55.6%)	543 (54.5%)	249 (58.2%)	
Yes	632 (44.4%)	453 (45.5%)	179 (41.8%)	
Marital status				0.637
Single	604 (42.4%)	427 (42.9%)	177 (41.4%)	
Married	820 (57.6%)	569 (57.1%)	251 (58.6%)	
Household income				0.701
<70000$	841 (59.1%)	592 (59.4%)	249 (58.2%)	
≥70000$	583 (40.9%)	404 (40.6%)	179 (41.8%)	

ATC, Anaplastic thyroid cancer; PSCCTH, Primary squamous cell carcinoma of thyroid; STNTT, Subtotal/Near-total Thyroidectomy.

We also performed chi-square tests between the training and validation cohorts, which showed no significant differences in the variables, indicating good comparability (**Table 1**).

### Overall survival and conditional survival

**Figure 1:**
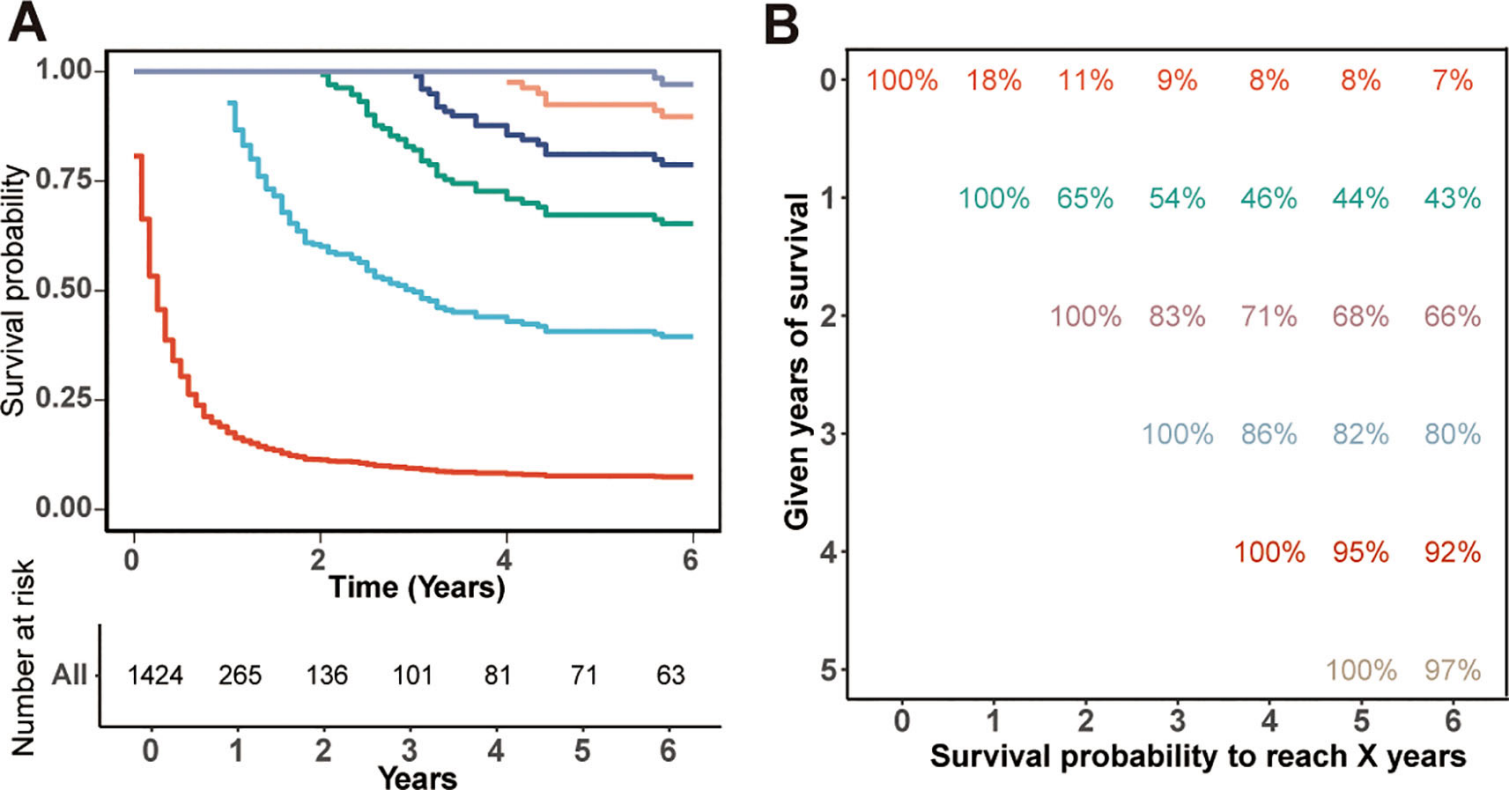
Kaplan-Meier estimates of survival post-diagnosis (0 years) and conditional survival, delineated by years already survived [1–4 years, **(A)**], along with the survival table **(B)**.

The OS rates for rATC patients were notably dismal, standing at only 18%, 9%, and 8% for the 1-, 3-, and 5-year intervals, respectively, with a vast majority of patients succumbing within the first year of diagnosis. The CS curves corresponding to the years survived after diagnosis are depicted in **Figure 1A**, while the CS probabilities are presented in **Figure 1B**. The probability of achieving 5-year CS increased from 8% immediately after diagnosis to 44%, 68%, 82%, and 95% given 1, 2, 3, and 4 years already survived, respectively. As patients survived longer, their probability of additional years of survival increased. However, this upward trend plateaued after 4 years of survival.

### Nomograms for patients who have survived for specific time

**Figure 2:**
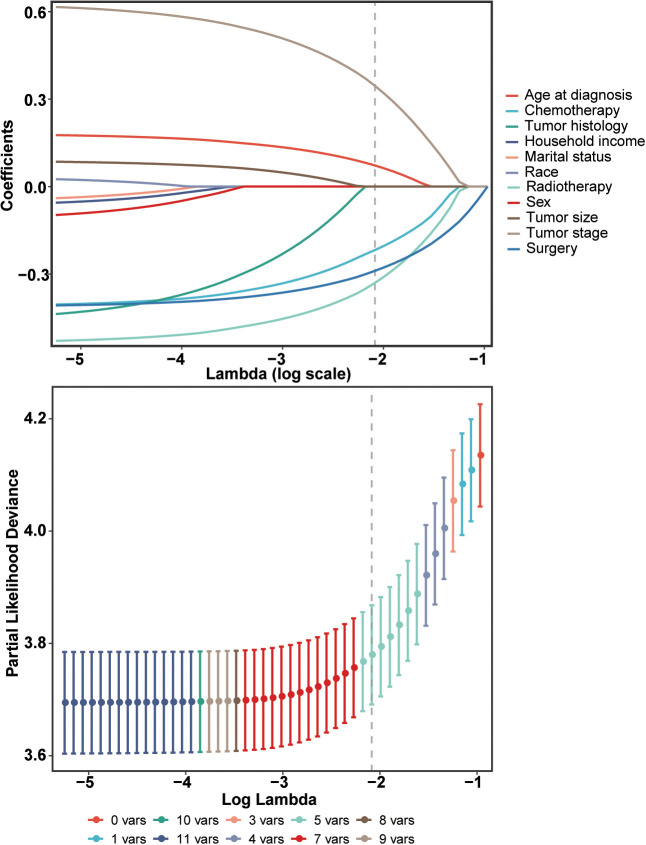
Identification of independent prognostic factors using a penalized least absolute shrinkage and selection operator (LASSO) regression model. This regularization technique was employed to select the most relevant prognostic variables while minimizing the risk of overfitting.

**Figure 3:**
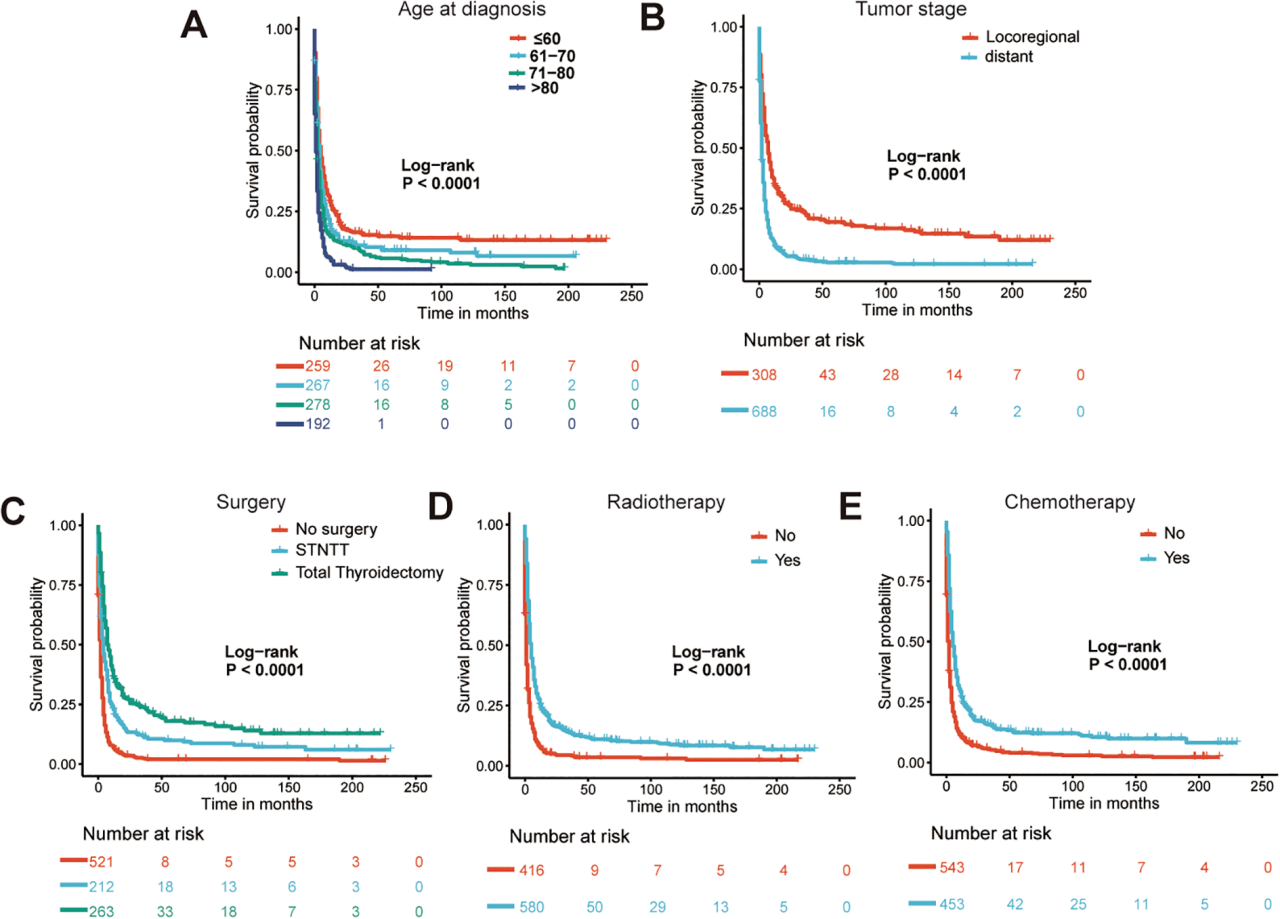
The Kaplan-Meier curves for prognostic indications of age **(A)**, tumor stage **(B)**, surgery **(C)**, radiotherapy **(D)** and chemotherapy **(E)**. STNTT, Subtotal/Near-total Thyroidectomy.

**Figure 4:**
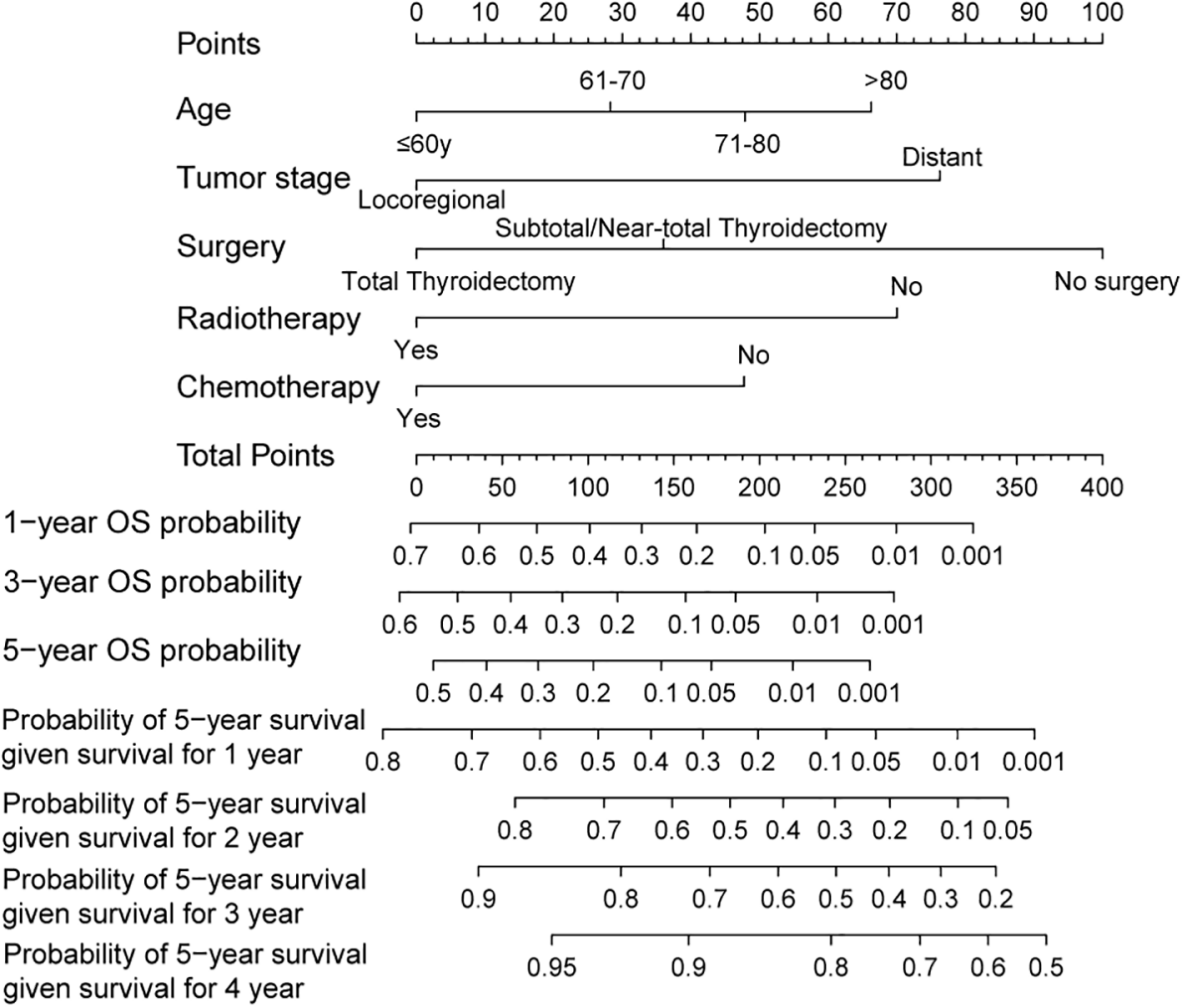
A conditional survival-based nomogram developed to predict the probability of 5-year conditional survival for individual patients based on significant prognostic variables. The model allows for dynamic survival prediction based on the number of years a patient has already survived post-diagnosis.

The Lasso Cox regression analysis was employed to identify the independent risk factors influencing the long-term survival of patients. In Lasso Cox analysis, each colored line in the model represents a variable. As λ increases, the coefficient of each variable decreases. At the optimal λ, some variables’ coefficients are shrunk to 0, allowing for the retention of non-zero variables and facilitating variable selection (**Figure 2**). Lambda 1se was selected for variable selection in our analysis, with the inclusion of age, tumor stage, surgery, radiotherapy, and chemotherapy deemed essential for constructing a prediction model. We further depicted Kaplan-Meier curves to validate the prognostic indications of age, tumor stage, surgery, radiotherapy and chemotherapy (**Figure 3**). Ultimately, we successfully established a novel CS-nomogram by integrating CS with a nomogram model based on patient clinical and pathological characteristics, aiming to provide dynamic survival updates and deeper prognostic insights for rATC patients (**Figure 4**).

### Nomogram evaluation and model-based risk stratification

**Figure 5:**
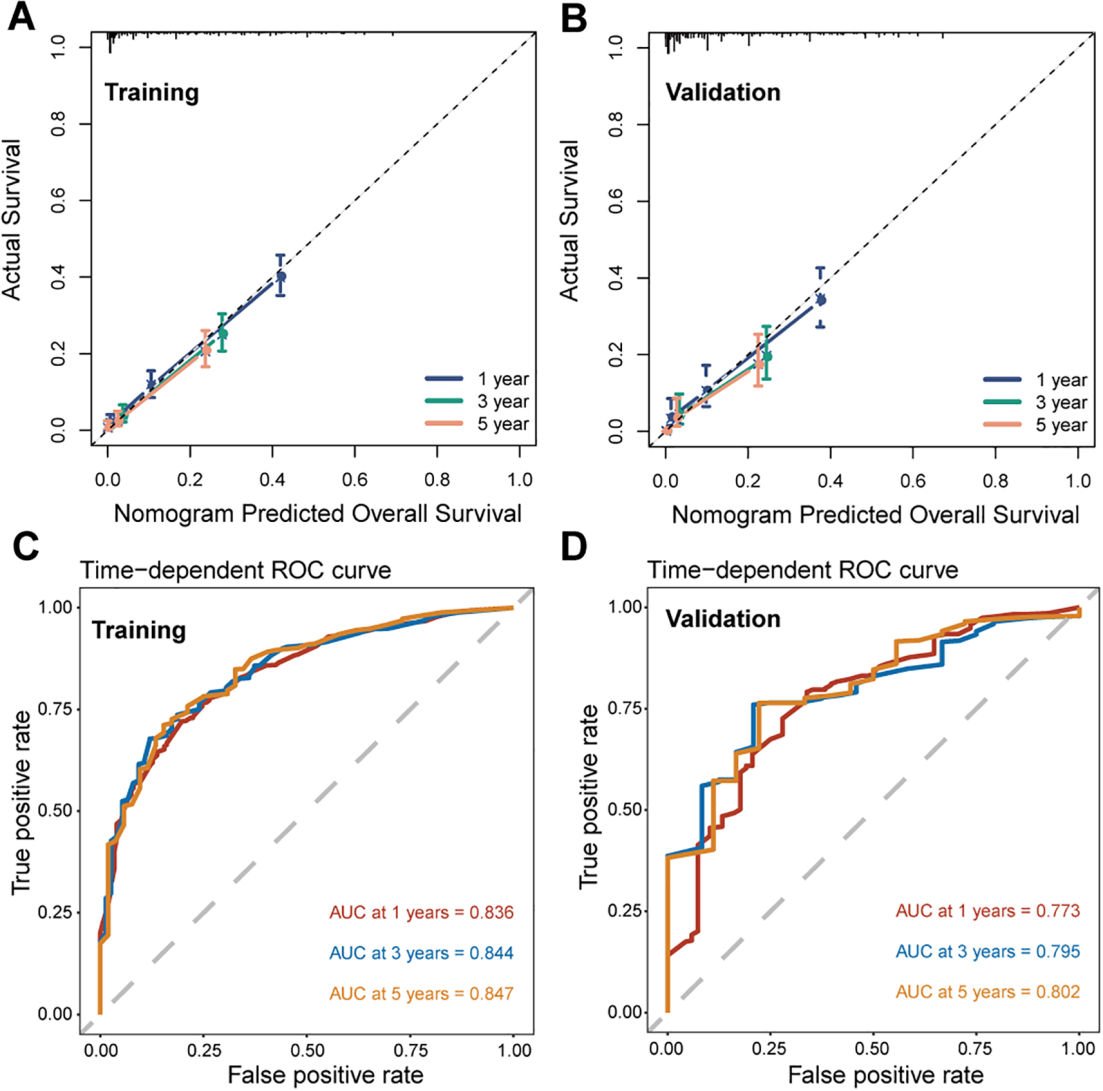
Performance evaluation of the conditional survival (CS) nomogram. Calibration curves for 1-, 3-, and 5-year survival probabilities are shown for the training cohort **(A)** and the validation cohort **(B)**, demonstrating the agreement between predicted and observed outcomes. Time-dependent receiver operating characteristic (ROC) curves and corresponding area under the curve (AUC) values for 1-, 3-, and 5-year survival are presented for the training **(C)** and validation **(D)** cohorts.

**Figure 6:**
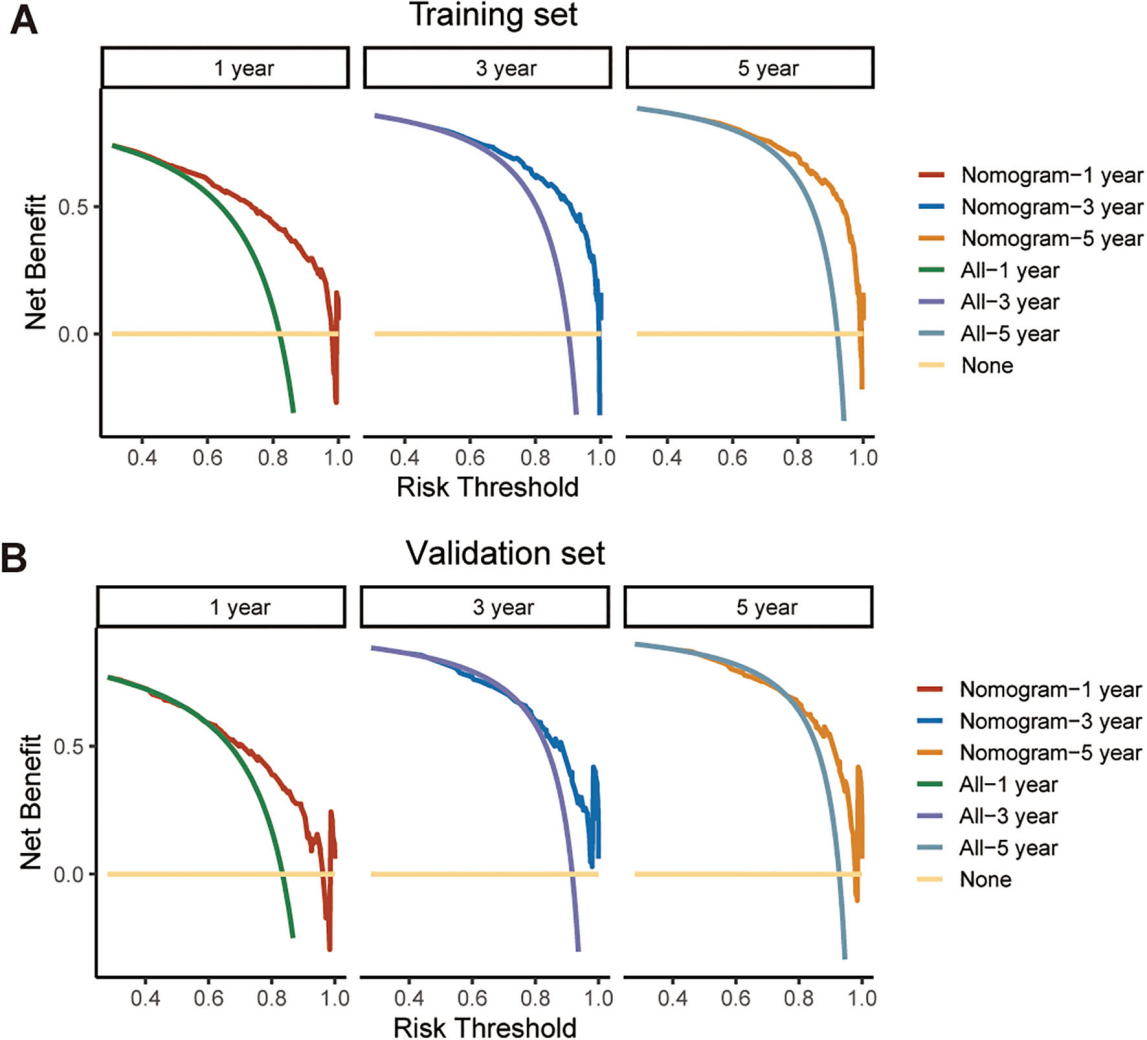
Decision curve analysis (DCA) for evaluating the clinical utility of the CS-nomogram in predicting survival at 1, 3, and 5 years. The net benefit across a range of threshold probabilities is shown for both the training cohort **(A)** and the validation cohort **(B)**, supporting the potential application of the model in clinical decision-making.

We further assessed the performance of the CS-nomogram. **Figures 5A, B** presents the calibration curves for the CS-nomogram applied to both the training and validation cohorts. These curves exhibit a robust agreement in forecasting survival probability, aligning closely with both predicted and observed survival rates within each cohort. The model’s discriminatory power was assessed using the C-index and ROC analysis with AUC values. The C-index, determined with 1000 bootstrap resampling, was 0.759 in the training cohort and 0.744 in the validation cohort, indicating robust predictive reliability. Meanwhile, the AUC of the CS-nomograms demonstrated a gradual increase with time. Within the training cohort, the nomogram exhibited AUC values of 0.836, 0.844, and 0.847 for 1, 3, and 5 years, respectively, whereas in the validation cohort, these values were 0.773, 0.795, and 0.802 (**Figures 5C, D**). These findings highlighted the nomogram’s robust discriminatory power and time-sensitive accuracy in predicting patient survival outcomes. According to **Figures 6A, B**, the DCA regarding the CS-nomogram model revealed satisfactory clinical net benefits over 1, 3, and 5-year horizons in both the training and validation cohorts. We further applied the model to two ATC patients (**Supplementary Figure 1**) from our center. Based on their clinicopathological characteristics, the calculated scores were 102 and 312, respectively. According to the nomogram, their predicted 1-, 3-, and 5-year survival probabilities were 40.0%, 25.0%, and 20.0% for the first patient, and 2.5%, <1%, and <1% for the second patient (**Table 2**). Using the CS-nomogram, conditional survival probabilities could be intuitively estimated, providing a dynamic and individualized assessment of survival over time. These results suggested that the CS-nomogram possesses good potential for practical application in clinical settings, as it can assist clinicians in making more informed, individualized prognostic assessments and treatment decisions, thereby improving patient management and long-term outcomes.

**Table 2:** Patient data included from our center.

Characteristics	Patient 1	Patient 2
Age		
≤60		
61-70		
71-80		
>80	✓	✓
Sex		
Male		
Female	✓	✓
Ethnicity		
White		
Black		
Others	✓	✓
Tumor Histology		
ATC	✓	✓
PSCCTh		
Tumor stage		
Locoregional	✓	
Distant		✓
Tumor size		
≤40mm		✓
41-60mm	✓	
>60mm		
Unknown		
Surgery		
No surgery		✓
Subtotal/Near-total Thyroidectomy	✓	
Total Thyroidectomy		
Radiotherapy		
No		✓
Yes	✓	
Chemotherapy		
No		
Yes	✓	✓
Marital status		
Single		
Married	✓	✓
Household income		
<70000$	✓	✓
≥70000$		
Score	102	312
predicted 1-year OS	40%	2.50%
predicted 3-year OS	25%	<1%
predicted 5-year OS	20%	<1%

ATC, Anaplastic thyroid cancer; PSCCTH, Primary squamous cell carcinoma of thyroid; STNTT, Subtotal/Near-total Thyroidectomy; OS, overall survival.

**Figure 7:**
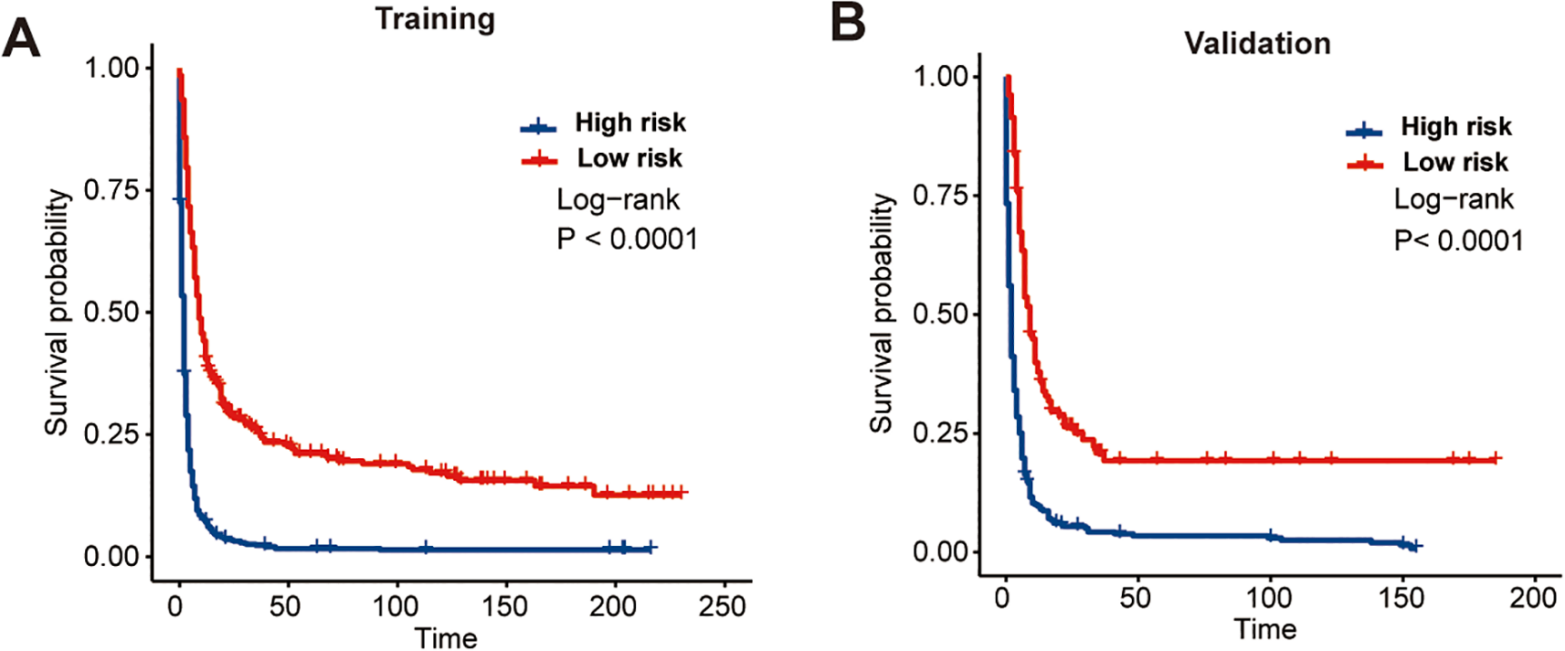
Kaplan-Meier survival curves demonstrating the effectiveness of the CS-nomogram in stratifying patients into distinct risk groups. Survival outcomes are shown separately for the training cohort **(A)** and the validation cohort **(B)**, confirming the model’s ability to distinguish between low- and high-risk populations.

Ultimately, the CS-nomogram was utilized to compute risk scores for each patient. Employing the optimal cutoff point (150 points) derived from calculations, we categorized patients into high-risk and low-risk groups. Subsequent survival analysis validated the risk stratification effect of the nomogram, demonstrating significantly better prognosis in the low-risk group compared to the high-risk group (**Figures 7A, B**).

## Discussion

Some nomograms have been devised to effectively assess the prognosis of individuals afflicted with ATC (9, 18, 20). Nonetheless, it is essential to acknowledge that the mortality risks subsequent to diagnosis are not constant. In cases where patients have survived beyond a certain period post-treatment, assessing prognosis solely based on OS at baseline may underestimate survival rates, necessitating frequent follow-up monitoring. This study investigated the CS outcomes of rATC patients and observed a significant enhancement in patients’ prognostic outlook with extended survival, notably among those who surpassed the first year post-diagnosis. For the first time, we devised a nomogram model that integrated CS analysis, aiming to furnish real-time, updated, and precise prognostic insights. This model can also facilitate the identification of high-risk populations and optimization of their follow-up strategies.

The nomogram model constructed using CS analysis presented in this study provides a refined prognostic estimation tailored to individual patients, thereby augmenting the efficacy of postoperative consultations. This heightened precision yields numerous advantages. It can serve as a corrective measure for adjusting survival prognoses among different patients, thus validating treatment efficacy. In clinical settings, it can also facilitate the identification of high-risk patients warranting more stringent follow-up plans. Furthermore, detailed prognostic information holds significant value for cancer patients facing poor prognoses. Increased prognostic probabilities calculated via CS-nomogram have the potential to profoundly influence the pessimistic mindset commonly observed among cancer patients.

Furthermore, within our model, the variables identified as most influential align consistently with clinical experience and corroborate findings from extant studies (18). The age, tumor stage, and treatment-related parameters, comprising surgical interventions, radiotherapy, and chemotherapy, were acknowledged as the predominant determinants exerting significant influence on long-term survival in our study. Surgery, radiotherapy, and chemotherapy have been identified as protective factors for survival in ATC patients, as supported by pertinent studies (4, 21, 22). Several studies have also reported that adjuvant therapies seemed to have no significant impact on prognosis following complete surgical resection (23–25). However, they may prove beneficial in cases of incomplete surgical resection or when surgery cannot be performed. Finally, we further validated the novel predictive model established based on these selected variables, confirming its high performance and demonstrating its potential clinical utility.

The primary strength of this study lies in its large sample size derived from a nationally representative population-based database, which is particularly valuable given the rarity of this disease. Such a robust cohort enhances the generalizability and statistical power of the findings. Moreover, the comprehensive and long-term follow-up data available in the SEER database allowed for high-quality input into the CS analysis, thereby increasing the reliability and clinical relevance of the results. Unlike traditional survival analysis, which provides static estimates from the time of diagnosis and may not accurately reflect prognosis at later time points, CS offers dynamic, real-time survival probabilities based on the duration a patient has already survived. This feature makes CS particularly valuable in clinical settings, where ongoing patient counseling and decision-making rely on up-to-date prognostic information. Finally, compared to previously established prognostic models, our CS-nomogram uniquely integrates CS functionality, enabling flexible and individualized survival predictions over time, and demonstrated favorable predictive accuracy and clinical utility.

This study also has several limitations. Firstly, it was conducted retrospectively, and due to incomplete data, some samples were excluded, resulting in a bias. Secondly, the database was found to be lacking several potential prognostic parameters, including performance status, comorbidities, and recurrence status. Thirdly, it did not offer specific details on surgical techniques, radiotherapy regimens, or chemotherapy protocols. Finally, despite the promising performance demonstrated in both the training and validation cohorts, this model still requires further external validation using independent, multicenter datasets. Such validation is essential to confirm its generalizability and applicability across diverse patient populations and clinical settings, ultimately enhancing its reliability as a tool for real-world clinical decision-making.

## Conclusion

Using a large, nationwide clinical dataset, this study has demonstrated the CS outcomes of patients with rATC, and we have also developed a CS-nomogram model to offer real-time updated precise predictions of long-term survival following diagnosis for these patients. Further external validation with additional data is also required.

## Data Availability

The original contributions presented in the study are included in the article/**Supplementary Material**. Further inquiries can be directed to the corresponding author.
