# Supplementary figures and images for: Evaluation of conditional survival outcomes in patients with redefined anaplastic thyroid carcinoma
Source: Front Endocrinol (Lausanne). 2025 Jun 10;16:1525869. doi: 10.3389/fendo.2025.1525869 (PMC12185289; doi:10.3389/fendo.2025.1525869)

The pathological diagnosis result of Patient 1.


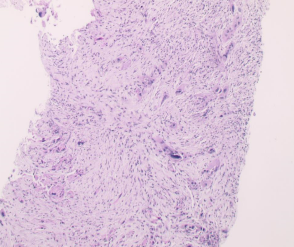

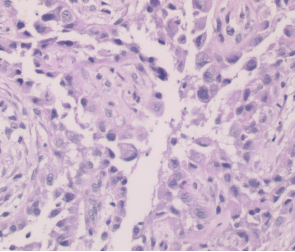


The pathological diagnosis result of Patient 2.


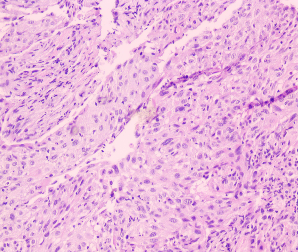

Supplement: Supplementary file 1 [file DataSheet1.docx]
